# Educational outcomes associated with childhood obesity in the United States: cross-sectional results from the 2011–2012 National Survey of Children’s Health

**DOI:** 10.1186/1479-5868-12-S1-S3

**Published:** 2015-07-27

**Authors:** Felicia R Carey, Gopal K Singh, H Shelton Brown III, Anna V Wilkinson

**Affiliations:** 1 Michael and Susan Dell Center for Healthy Living, The University of Texas School of Public Health Austin Regional Campus, Austin, TX, 78701, USA; 2 U.S. Department of Health and Human Services, Health Resources and Services Administration, Maternal and Child Health Bureau, Rockville, MD, 20857, USA

**Keywords:** Childhood obesity, social determinants, educational-outcomes, United States

## Abstract

**Background:**

Past research examining the effects of childhood obesity has largely focused on its projected effects into adulthood. However, there is emerging evidence that childhood obesity may have more immediate effects on school-related outcomes. We examine a range of educational attainment indicators to examine the possible pathway between obesity status and academic performance, while investigating the proximal effects of childhood obesity on health and utilization of health services, and whether these variables attenuate the relationship between obesity status and educational outcomes.

**Methods:**

Data for the current study come from the 2011-2012 National Survey of Children’s Health, which details the impacts of childhood obesity on a range of outcomes among a nationally representative sample of children and adolescents aged 10-17 years (N=45,255). Educational outcomes (school absences, school problems, repeating a grade and school engagement) were modeled by logistic regression as a function of BMI, overall health status, health care utilization, and a range of sociodemographic variables.

**Results:**

BMI status was significantly associated with all educational outcomes (p<0.001 for all), overall health status (p<0.001), and health care utilization (p=0.016). Prior to adjustment for covariates, obese children were significantly more likely to have school absences and school problems, to repeat a grade, and to have lower school engagement than non-overweight children. After adjustment for sociodemographic and health/healthcare variables, these outcomes remained significant for all but repeating a grade. The odds of having school problems, repeating a grade, and low school engagement that were associated with obesity were attenuated by the addition of sociodemographic variables into the model, while the addition of health and health care variables in the model decreased the odds of school absences.

**Conclusions:**

This study provides evidence that increased weight status in children is associated with poorer educational outcomes. While recognizing that these are cross-sectional data, we suggest that 1) health-related and sociodemographic factors should be a focus point of intervention, and 2) a socio-structural approach including Coordinated School Health intervention is crucial to reducing childhood obesity and improving educational outcomes in this population.

## Background

Over the last three decades, there has been a tremendous increase in the number of overweight and obese children in the US [1-3] and worldwide [4]. While some have projected the lifetime economic and health impacts of obesity [5], less is known about the more immediate impacts of obesity on children and adolescents. Until recently, when the effects of childhood obesity were studied, emphasis was more heavily placed on its projected effects into adulthood and the health effects of obesity, rather than on other outcomes. However, there is emerging evidence that childhood obesity may have more immediate, direct effects as well, specifically on school and health related outcomes, which themselves can, in turn, have lifetime effects.

Evidence from both cross-sectional and longitudinal studies suggests that obese children encounter more behavioral problems in school than non-obese children, such as internalizing problems (e.g., low self-esteem, sadness, acting withdrawn), externalizing problems (e.g., arguing, fighting, disobedience), and school discipline problems (e.g., detentions and suspensions) [6-8], with the presence of these behavioral problems increasing significantly with increased weight status [9]. Likewise, academic problems are common among obese adolescents. As compared to their normal weight peers, obese children are significantly more likely to repeat a grade in school [7,10,11], a sign of inadequate academic achievement in the United States that indicates that the student failed to gain the educational or social skills expected of them upon completion of their current grade level [12]. Obese adolescents are absent from school more often than their normal weight counterparts [7,8,10,13], with absenteeism showing a gradient with obesity such that adolescents in the highest weight status groups are absent at significantly higher rates [14,15]. Overall school engagement is lower in obese adolescents as well [10], and academic effort decreases significantly as weight status increases [16]. Some of the effects of weight status on academic outcomes also may be more pronounced in girls than in boys [6,11].

In many studies, obese adolescents report lower general health scores [17,18], lower health-related quality of life [19], and higher rates of comorbid conditions [7]. In addition, children and adolescents with higher body mass index (BMI) report greater outpatient and emergency room visits, as well as significantly higher health care expenditures in both areas, as compared to children of normal weight [20,21]. Thus, the adverse effects associated with obesity not only potentially increase health care costs, but the increased utilization of health care services may necessitate school absences to address these issues, leading also to real effects on educational attainment and achievement during teenage years and into young adulthood. Such childhood effects may continue into adulthood, placing obese children at a disadvantage in terms of college attendance and subsequent options for future employment [22].

While obesity could cause poor academic outcomes, there is the potential for reverse causality between academic performance and weight status [6]. Even though establishing the direction of causality is beyond the scope of our paper, we suggest and examine the following pathway: poorer school-related outcomes experienced by obese children could be a function of their aforementioned poorer overall physical health and associated higher utilization of health services. Thus, in the current paper, we present results from the 2011-2012 National Survey of Children’s Health, which details the relationship between childhood obesity and a wide range of outcomes among a nationally representative sample of children and adolescents aged 10-17 years. We first examine a range of educational attainment indicators which include days absent, promotion to subsequent grade, problems at school, and school engagement by three weight-status groups: non-overweight, overweight, and obese. Second, we investigate the proximal effects of childhood obesity on parent-reported child health and utilization of health care services by their children, both of which could increase days absent from school and/or negatively impact academic performance, also compared by the three weight-status groups. Finally, we examine whether parent-reported child health status attenuates the relationship between obesity status and number of days absent from school, promotion to the subsequent grade, experiencing school-related problems, and not being engaged in school, as this would provide preliminary evidence for a causal pathway between obesity status and academic performance. Even if the association between obesity status and academic performance is biased upward due to reverse causality (i.e., children in poor physical health may have increased body weight), our results would provide early estimates from a very large sample about a potential causal path.

## Methods

Data for the present study came from the 2011-2012 National Survey of Children’s Health (NSCH) [23,24]. The survey was conducted in the U.S. by the National Center for Health Statistics (NCHS), with funding and direction from the Maternal and Child Health Bureau [23,24]. The purpose of the NSCH was to provide national and state-specific prevalence estimates for a variety of children’s health and well-being indicators. The survey included an extensive array of questions about children’s health and the family, including parental health, stress and coping behaviors, family activities, and parental concerns about their children [23,24]. Interviews were conducted with parents, and special emphasis was placed on factors related to children’s well-being.

The 2011-2012 NSCH was a cross-sectional telephone survey conducted between February 2011 and June 2012 [23,24]. The two previous rounds of the NSCH were conducted in 2003-2004 and 2007-2008 [24-27]. The 2011-2012 survey had a sample size of 95,677 children <18 years of age, including a sample of >1,800 children per state [23,24]. In the survey, a random-digit-dial sample of households with children <18 years of age was selected from each of the 50 states and the District of Columbia. One child was selected from all children in each identified household to be the subject of the survey [23,24]. Interviews were conducted in English, Spanish, and four Asian languages. The respondent was the parent or guardian who knew most about the child’s health status and health care. All survey data, including height and weight to calculate body mass index (BMI), were based on parental reports. The interview completion rate for the 2011-2012 NSCH, a measure of the response rate indicating the percentage of completed interviews among known households with children, was 54.1% for the landline sample and 41.2% for the cell-phone sample [23,24]. Substantive and methodological details of the 2011-2012 NSCH are described elsewhere [23]. The NCHS Research Ethics Review Board approved all data collection procedures for the survey.

The sample size for the present analysis was 45,255 children and adolescents aged 10-17 years. Associations between child’s BMI status and six educational and health outcomes were assessed. Educational outcomes included school absence, child having a problem at school, child repeating a grade, and school engagement [23]. School absence was based on the question, “During the past 12 months, about how many days did the child miss school because of illness or injury?” To be consistent with past methods for analysis of the variable [27], school absence was dichotomized, with children missing more than 2 weeks (>10 days) of school being classified as “1”=yes and “0” otherwise. “School problems” was derived from the question, “During the past 12 months, how many times has the child’s school contacted the parent or another adult in household about any problem the child is having with school?”, with any positive response categorized as “yes”, and no reported contact by the school categorized as “no”. “Repeating a grade” was based on the question that asked parents if their children had repeated one or more grades since starting school. School engagement was derived from two questions that asked parents if their child cares about doing well in school and whether the child does all required homework. Children were considered to have low school engagement if their parents responded “never, rarely, or sometimes” to both of these items.

Parent-reported overall health status of the child was dichotomized into two categories: excellent/very good/good and fair/poor. Child’s BMI status, as determined by parent-reported height and weight of the child, was the primary covariate of interest and consisted of three categories: non-overweight (BMI<85th percentile), overweight (85th≤BMI<95th percentile), and obese (BMI≥95th percentile) [2,3]. Healthy weight (5th≤BMI<85th percentile) and underweight (BMI<5th percentile) children were combined into a single category due to the low prevalence of underweight children in the sample (<2%).

Using the social-determinants-of-health framework, in which sociodemographic and socio-structural factors act as underlying upstream factors to influence obesity primarily through their effects on individual behaviors, such as diet, physical activity, and sedentary behavior [28-31], and past research as a guide, we considered a wide range of covariates. In addition to BMI, we examined the following covariates of educational and health outcomes: child’s age, gender, race/ethnicity, household composition, metropolitan/non-metropolitan residence, household/parental education level, and household poverty status measured as a ratio of family income to the poverty threshold [24,32-34]. The selection process and measures for these covariates have been described in detail elsewhere [26]. Since we hypothesized that the association between BMI status and school outcomes could be attenuated by health status and/or healthcare utilization, we considered overall health and healthcare visits as intervening variables when modeling determinants of school outcomes. The health-care visit variable was defined if the child visited a doctor or healthcare provider in the past year for well- or sick-care (i.e. preventive health services as well as treatment). These covariates were measured as shown in Tables 1-2.

**Table 1:** Descriptive statistics of the sample, according to sociodemographic and health characteristics (N = 45,255)

Sociodemographic characteristics	Unweighted number in sample	Weighted percent in sample
Child’s BMI status		
Non-overweight (<85th percentile)	31076	68.7
Overweight (85th to <95th percentile)	6495	15.6
Obese (≥95th percentile)	6293	15.7
Child’s age (years)		
10-12	10692	24.6
12-14	16145	37.2
15-17	18418	38.2
Child’s gender		
Male	23597	51.2
Female	21658	48.8
Race/ethnicity		
Hispanic	5213	20.8
Non-Hispanic white	30488	53.9
Non-Hispanic black	4260	14.0
Non-Hispanic mixed race	2165	4.4
Other (Asian/Pacific Islanders and American Indians)	3129	6.9
Household composition		
Two-parent biological	29342	58.5
Two-parent stepfamily	4543	12.8
Single mother	7395	19.6
Other family type	3975	9.1
Place of residence		
Metropolitan	33051	83.9
Non-metropolitan	11663	16.1
Highest household or parental education level (years)		
<12	2477	11.5
12	6994	19.8
13-15	11427	24.8
16+	23255	43.9
Household poverty status (ratio of family income to poverty threshold)		
Below 100%	5993	19.8
100-199%	7725	21.3
200-399%	14079	28.8
At or above 400%	17458	30.1
Child’s overall health status		
Excellent/very good/good	43945	96.5
Fair/poor	1294	3.5
Child’s visit to a doctor/health care provider for well- or sick-care during past year		
Yes	39720	86.0
No	5448	14.0
Child missed >2 weeks of school during a year		
Yes	2893	6.6
No	40583	93.4
Child reported having a problem at school		
Yes	12393	31.1
No	30069	68.9
Child repeated a grade		
Yes	3741	10.6
No	40011	89.4
Child reported low school engagement		
Yes	9468	22.4
No	34291	77.6

**Table 2:** Weighted prevalence of selected educational outcomes among the sample by BMI, sociodemographic, and health characteristics (N = 45,255)

Sociodemographic characteristics	School absence (missing >2 weeks of school in past year)			Problem at school (child reported having a problem at school)			Child repeated a grade in school			Child reported low school engagement			Fair/poor overall health			Healthcare visits		
	%	SE	P-value	%	SE	P-value	%	SE	P-value	%	SE	P-value	%	SE	P-value	%	SE	P-value
Child’s BMI status			<0.001			<0.001			<0.001			<0.001			<0.001			0.016
Non-overweight	6.1	0.33		28.3	0.61		9.6	0.41		20.7	0.53		2.4	0.24		87.1	0.48	
Overweight	5.9	0.61		36.0	1.39		11.7	0.95		23.9	1.18		3.3	0.42		88.1	0.88	
Obese	9.8	0.86		38.5	1.43		13.6	0.89		28.0	1.27		7.0	0.72		83.7	1.27	
Child’s age (years)			<0.001			0.009			<0.001			<0.001			0.125			0.069
10-11	4.8	0.53		31.5	1.07		9.2	0.71		16.9	0.85		3.3	0.50		84.8	0.94	
12-14	6.2	0.41		32.9	0.86		10.0	0.54		21.7	0.72		3.0	0.33		87.2	0.63	
15-17	7.8	0.47		29.3	0.82		12.5	0.60		26.2	0.77		4.0	0.32		85.7	0.66	
Child’s gender			0.833			<0.001			<0.001			<0.001			0.378			0.020
Male	6.5	0.38		37.6	0.76		12.9	0.52		29.4	0.70		3.6	0.34		85.1	0.61	
Female	6.4	0.38		24.4	0.69		8.5	0.46		14.7	0.53		3.3	0.25		87.0	0.56	
Race/ethnicity			<0.001			<0.001			<0.001			<0.001			<0.001			<0.001
Hispanic	6.1	0.82		33.3	1.53		12.3	1.05		20.6	1.32		7.1	0.84		80.4	1.30	
Non-Hispanic white	7.4	0.34		27.8	0.58		8.4	0.35		21.4	0.51		1.9	0.14		89.4	0.42	
Non-Hispanic black	4.4	0.54		43.1	1.42		19.3	1.15		30.5	1.28		4.7	0.58		84.3	1.01	
Non-Hispanic mixed race	8.7	1.06		37.7	2.38		11.1	1.37		21.5	1.67		3.4	0.72		89.0	1.23	
Other	2.9	0.46		22.4	1.72		6.9	1.26		17.3	1.75		2.0	0.36		78.2	2.13	
Household composition			<0.001			<0.001			<0.001			<0.001			<0.001			0.001
Two-parent biological	5.3	0.32		25.2	0.64		6.7	0.36		16.1	0.49		2.7	0.27		87.1	0.53	
Two-parent stepfamily	7.2	0.75		36.6	1.53		14.5	1.13		31.3	1.48		4.0	0.71		84.7	1.29	
Single mother	9.4	0.68		41.3	1.24		17.9	1.01		30.3	1.11		5.2	0.52		86.4	0.85	
Other family type	6.5	1.07		40.1	1.87		16.4	1.35		31.5	1.84		3.5	0.49		80.3	1.67	
Place of residence			0.074			0.291			<0.001			0.537			0.166			0.005
Metropolitan	6.3	0.31		31.2	0.59		9.9	0.38		22.1	0.51		3.5	0.25		86.6	0.47	
Non-metropolitan	7.6	0.54		30.2	1.00		14.8	0.89		23.2	0.84		2.9	0.28		83.4	0.90	
Highest household/parental education			0.005			<0.001			<0.001			<0.001			<0.001			
<12 years	6.3	0.77		38.7	2.09		20.8	1.69		25.6	1.79		9.5	1.23		74.8	1.81	
12 years	7.2	0.63		35.3	1.24		15.9	0.90		27.4	1.07		4.4	0.47		81.1	1.08	
13-15 years	7.9	0.61		35.1	1.06		12.6	0.75		26.0	0.94		3.2	0.30		86.2	0.80	
16+ years	5.4	0.38		25.0	0.66		4.7	0.31		16.7	0.57		1.6	0.25		91.6	0.48	
Household poverty status			<0.001			<0.001			<0.001			<0.001			<0.001			<0.001
Below 100%	8.4	0.69		39.4	1.48		22.0	1.20		29.2	1.28		7.6	0.68		77.6	1.27	
100-199%	7.2	0.54		36.4	1.30		13.0	0.79		24.5	1.04		4.4	0.59		82.0	1.06	
200-399%	6.4	0.54		30.1	0.94		7.9	0.55		21.6	0.81		2.6	0.38		87.7	0.72	
At or above 400%	4.8	0.44		23.2	0.78		4.6	0.35		16.7	0.71		0.9	0.12		92.9	0.50	
Child’s overall health status			<0.001			<0.001			<0.001			<0.001						
Excellent/very good/good	5.7	0.26		30.4	0.52		10.3	0.35		21.7	0.45							
Fair/poor	29.3	2.69		53.6	3.25		24.0	2.61		38.6	3.04							
Healthcare visit for well- or sick-care			<0.001			<0.001			0.004			0.434						
Yes	7.0	0.30		32.0	0.55		10.2	0.35		22.1	0.47							
No	2.9	0.42		26.2	1.55		14.0	1.24		23.3	1.45							

Note: p-values are associated with chi-square tests for independence between each covariate and educational or health outcome.

Income was estimated using a multiple imputation technique [23,26] for the 9% of participants with missing income data. However approximately 3.1% of the participants aged 10-17 had missing BMI data; their data were excluded from the analysis. For all other covariates and outcome variables, there were few or no missing cases; these were excluded from the multivariate analyses, yielding an effective sample size of at least 43,600 for most of the analyses.

The χ^2^ statistic was used to test the overall association between covariates and each outcome. The t-statistic was used to test the difference in prevalence between any two groups. Logistic regression was used to examine the association between BMI status and educational or health outcomes, after adjusting for the aforementioned covariates. To account for the complex sample design of the NSCH, Survey Data Analysis (SUDAAN) software was used to conduct all statistical analyses [35]. Methodology used for computing sample weights has been previously detailed [26], and resulting weighted estimates are representative of all non-institutionalized children in the US at the national level.

## Results

Sociodemographic and health characteristics of the study population (n=45,255) are presented in Table 1. Among the study population, 68.7% (n=31,076) were non-overweight, 15.6% (n=6,495) were classified as overweight, and 15.7% (n=6,293) were classified as obese. In regards to health characteristics, only 3.5% (n=1,294) of children were reported to be in fair or poor overall health and 86.0% (n=39,720) of children were reported having visited a doctor or health care provider for well- or sick-care in the past year. Overall, 6.6% (n=2,893) of children were reported as having missed more than two weeks of school, 31.1% (n=12,393) were reported as having school problems, 10.6% (n=3,741) were reported as having repeated a grade, and 22.4% (n=9,468) of children were reported as having low school engagement.

The weighted prevalence of selected educational and health outcomes of the study population are presented in Table 2. All educational outcomes were significantly associated with BMI status (p<0.001 for all). School problems displayed a positive association with increasing BMI status, with 36.0% of overweight children reported having a problem at school in comparison with 28.3% of their non-overweight peers, and obese children showing the highest prevalence of school problems at 38.5%. Repeating a grade and low school engagement displayed similar associations, with 13.6% of obese children having repeated a grade in comparison to 11.7% of overweight children and 9.6% of non-overweight children, and 28.0% of obese children reporting low school engagement in comparison to 23.9% of overweight children and 20.7% of non-overweight children. School absences did not display the same linear trends; obese children had a higher prevalence of missing >2 weeks of school in the past year (9.8%) than did their non-overweight peers (6.1%), while overweight children had the lowest prevalence of school absences (5.9%).

BMI status was also significantly associated with health status and health care utilization in the study population. Poor health status was positively associated with BMI status (p<0.001), with 7.0% of obese children, 3.3% of overweight children, and 2.4% of non-overweight children being in fair or poor overall health. The association between healthcare visits and BMI was significant at p=0.016, with obese children (87.1%) and overweight children (88.1%) being more likely to have visited healthcare providers in the past year (83.7%) than non-overweight children (87.1%).

Overall health status was significantly associated with all educational outcomes (p<0.001 for all). Children in fair or poor overall health also had a higher prevalence of school absences, problems at school, repeating a grade, and low school engagement. Of the educational outcomes, healthcare visits were significantly associated with school absences (p<0.001), school problems (p<0.001), and repeating a grade (p=0.004). Children who had one or more health care visits for well- or sick-care in the past year had a higher prevalence of school absences (7.0%) and school problems (32.0%) than those who did not have health care visits (2.9% and 26.2%, respectively). On the other hand, children who had one or more health care visits in the past year had a lower prevalence of repeating a grade (10.2%) than did children with no health care visits (14.0%).

Overweight (OR=1.41; 95% CI: 1.02-1.96) and obese (OR=3.11; 95% CI: 2.31-4.18) children were significantly more likely to be in fair or poor overall health status than non-overweight children prior to adjustment for other covariates (data not shown). Obese children only were significantly less likely to have one or more health care visits within the past year (OR=0.76; 95% CI: 0.62-0.93) than non-overweight children. After adjustment for sociodemographic variables, obese children were still more likely to be in fair or poor overall health (OR=2.25; 95% CI: 1.62-3.12) than non-overweight children. In contrast, health care visits became significantly associated with overweight status, with overweight children being more likely to have at least one health care visit in the past year (OR=1.31; 95% CI:1.09-1.59) than non-overweight children, while the association between health care visits and obese weight status did not reach statistical significance.

Logistic regression analyses of BMI and selected educational outcomes are presented in Table 3. Prior to adjustment for other covariates, obese children were significantly more likely to have school absences and school problems, repeat a grade, and report low school engagement than non-overweight children (Model I). Overweight children were significantly more likely to have school problems, repeat a grade, and report low school engagement than non-overweight children (Model I). After adjustment for sociodemographic variables, obese children were significantly more likely to have school absences and school problems, and report low school engagement compared to non-overweight children, while overweight children were only significantly more likely to have school problems (Model II). After full adjustment for both the sociodemographic and health/healthcare variables, obese children remained significantly more likely to have school absences (OR=1.49), school problems (OR=1.17), and low school engagement (OR=1.18) than non-overweight children; overweight children were significantly more likely to have school problems (OR=1.22) than non-overweight children (Model III).

**Table 3:** Unadjusted and adjusted logistic regression analyses of BMI and selected educational outcomes among the sample

	School Absences								
	Model I			Model II			Model III		
BMI	OR	95% CI	P-value	OR	95% CI	P-value	OR	95% CI	P-value
Overweight (85th to <95th percentile)	0.97	0.76-1.23	0.783	0.98	0.76-1.27	0.882	0.94	0.72-1.22	0.628
Obese (≥95th percentile)	1.69	1.36-2.11	<0.001	1.67	1.29-2.16	<0.001	1.49	1.13-1.96	0.005
	School Problems								
	Model I			Model II			Model III		
BMI	OR	95% CI	P-value	OR	95% CI	P-value	OR	95% CI	P-value
Overweight (85th to <95th percentile)	1.42	1.24-1.62	<0.001	1.24	1.08-1.43	0.002	1.22	1.06-1.40	0.006
Obese (≥95th percentile)	1.58	1.38-1.80	<0.001	1.21	1.05-1.40	0.007	1.17	1.01-1.35	0.031

	Grade Repeated								
	Model I			Model II			Model III		
BMI	OR	95% CI	P-value	OR	95% CI	P-value	OR	95% CI	P-value
Overweight (85th to <95th percentile)	1.24	1.01-1.52	0.037	1.01	0.81-1.25	0.946	1.01	0.81-1.25	0.959
Obese (≥95th percentile)	1.48	1.24-1.76	<0.001	0.97	0.80-1.17	0.720	0.94	0.78-1.14	0.548

	Low School Engagement								
	Model I			Model II			Model III		
BMI	OR	95% CI	P-value	OR	95% CI	P-value	OR	95% CI	P-value
Overweight (85th to <95th percentile)	1.20	1.05-1.39	0.044	1.10	0.95-1.28	0.215	1.10	0.94-1.28	0.222
Obese (≥95th percentile)	1.49	1.30-1.71	0.261	1.21	1.04-1.41	0.014	1.18	1.02-1.38	0.032

Notes: OR = odds ratio. CI = confidence interval. Reference category for BMI is non-overweight (<85th percentile).

a. Model I: unadjusted for the other covariates.

b. Model II: adjusted for sociodemographic characteristics including age, gender, race/ethnicity, household composition, place of residence, parental education, and household poverty level.

c. Model III: fully adjusted for all sociodemographic and intervening variables, including overall health status and healthcare visits. The full models for repeated grade and school engagement exclude healthcare visits.

## Discussion

Consistent with previous literature [7,8,10,13], our findings revealed that obese children had the highest prevalence of school absences of any weight group. However, unlike previous findings which showed that school absences increased as weight status increased [14,15], we found that both non-overweight and overweight adolescents had comparable but lower prevalence rates of absences compared to obese adolescents, and obese children were significantly more likely to miss 10 or more days of school compared to their overweight or non-overweight peers. Controlling for sociodemographic variables had little effect on the likelihood of school absences among obese children, while the addition of health and health care variables in the model decreased the likelihood. This suggests that increased absences in school are directly associated with weight status among obese children and adolescents only, and that poor overall health and/or health care visits may account for some of the days that they are absent from school.

With regards to school problems [6-9], repeating a grade [7,10,11], and school engagement [10], our results were also consistent with previous literature, with obese children having the highest prevalence of school problems, repeating a grade, and low school engagement. Overweight and obese children were significantly more likely to experience problems in school, even after adjustment for sociodemographic and health/healthcare variables, though odds ratios in both weight status categories were reduced further by the addition of sociodemographic variables into the model than by health/healthcare variables. In unadjusted models, both overweight and obese children were more likely to repeat a grade than non-overweight children. However, after adjustment for sociodemographic variables, this association was not significant for either overweight or obese children, and remained non-significant after further adjustment for health status and healthcare utilization. Similar to school problems and repeating a grade, the odds of not being engaged in school were reduced by the addition of sociodemographic variables into the model, suggesting that in obese children these three educational outcomes are more closely associated with the children’s sociodemographic characteristics rather than with health status or healthcare utilization.

The prevalence of overweight (15.6%) and obesity (15.7%) among our study sample revealed little change from the 2007-2008 NSCH (overweight: 15.2%, obesity: 16.4%) [3], and is consistent with trends among both the National Health and Nutrition Examination Survey (NHANES) [36] and the US Health Behavior in School Aged Children (HBSC) Study [37]. This seems to indicate a leveling of the trend in US childhood obesity after three decades of marked and consistent increases [1-3].

In general, our results present stronger evidence for an association between BMI status and educational outcomes. However, we note a significant attenuation of the association with educational outcomes among obese and overweight children when considering sociodemographic or health/healthcare variables, or a combination of both. Consistent with previous research [6,38], sociodemographic outcomes, such as household composition, parental education, and poverty level, were all independently and significantly associated with all educational outcomes, in all of the fully adjusted models (data not shown).

In relation to health outcomes, overall health status was significantly associated with each of the four educational outcomes, while healthcare visits were significantly associated with school absences, school problems, and repeating a grade. Both health outcomes were also significantly associated with BMI status. After adjustment for sociodemographic variables, obesity status remained significantly associated with overall health status, but was no longer significant for health care visits. In contrast, overweight adolescents became significantly more likely to utilize health care visits after adjustment. This suggests that in the case of overweight adolescents, sociodemographic attributes may act as a barrier to seeing a health care provider, while obese adolescents are less likely to see doctors for other reasons – perhaps because of an aversion to receiving health care due to stigmatization based on their weight and subsequent poor treatment by health care providers [39]. Other factors may influence health care utilization within the sample as well. For instance, a higher percentage of obese and overweight children in the sample did not have access to a medical home – defined as an established team of providers that meet a child’s medical and non-medical care needs – as compared to normal weight children, and obese adolescents were more often uninsured or insured through government and state sponsored programs (i.e. Medicaid/SCHIP) rather than private health insurance [23]. We also had no ability to account for use of school-based health centers in our study population, the presence of which is more common in areas with populations who are prone to a higher prevalence of obesity, such as ethnic minorities or low socioeconomic status populations [40].

Based on the associations presented within our results, we suggest two main areas of intervention to improve educational outcomes in overweight and obese youth: 1) interventions that target proximal variables, such as reducing doctor’s visits through improvement of overall health status, by focusing on improvement of individual behaviors like nutrition and physical activity, and 2) policy and socio-structural level interventions that focus on reducing socioeconomic inequalities that are associated with these outcomes.

Interventions targeted to improving the health status of overweight and obese children have been studied extensively in the past [41,42]. These interventions, which often include the introduction of nutritional and behavioral change programs into schools and strategies to improve access to healthy food, increase physical activity levels, and reduce sedentary behaviors in adolescents, have achieved some level of efficacy in both improving adolescent health and reducing BMI in the target populations. It is important to recognize that while individual level interventions have been effective in this area [43], the success of such interventions is localized and may have limited success on a broader scale when not implemented in combination with societal level interventions. Without such societal level change, these interventions in fact may only motivate those who are more socially advantaged, and thus may worsen disparities between advantaged and disadvantaged adolescents [44]. As such, the sociodemographic variables identified in this study that place adolescents at a disadvantage should also to be targeted for intervention. One of the more frequently cited causes of poor health in disadvantaged children is a lack of access to health insurance; however, this is less of the issue than is access to proper health care itself [45]. This further underscores the need to take a structural approach to addressing issues related to poverty for disadvantaged children.

Efforts to improve the health and educational outcomes of obese children and adolescents, especially those of a lower socioeconomic status, are particularly important in light of the economic link to school outcomes and obesity. Having a lower socioeconomic status is linked to poorer school outcomes and obesity in adolescence, which in turn are linked to reduced educational and work opportunities in the future [46], thus resulting in maintenance of low socioeconomic status into adulthood and perpetuating the cycle for future generations. The findings from our study, while needing further verification with longitudinal data, are in line with this premise and do suggest that some of the long-term costs of childhood obesity may result from early school and health problems.

Given that childhood obesity can be reduced in part by increasing physical activity and promoting good nutrition among adolescents [41,42], implementing effective interventions that both promote nutrition and physical activity and also address socioeconomic inequalities associated with poor health and educational outcomes is essential. We suggest the school itself as the ideal setting for this type of intervention. Schools in the U.S. already address issues related to nutrition and socioeconomic inequality through programs sponsored by the United States Department of Agriculture (USDA), such as the National School Lunch Program (NSLP), which provided nutritionally balanced, free or low-cost meals to more than 31.6 million children from low-income families in 2012 alone [47]. However, implementation of more comprehensive school based programs are necessary to address the variety of issues affecting the health and educational outcomes of children and adolescents in the U.S. Along these lines, the Centers for Disease Control and Prevention (CDC) provides recommendations for a Coordinated School Health approach, which incorporates health and physical education into student curricula, promotes the use of school based health, mental health, and social services, incorporates nutrition services into the school environment, promotes faculty and staff wellness, and seeks to increase family and community involvement [48]. This framework for school health promotion has achieved some level of success in reducing the prevalence of obesity and overweight, increasing physical activity levels, and promoting better nutrition among school populations [48]. Additionally, the use of school based health centers (SBHC) has been shown to improve the quality of care received and provide easier access to health care, especially preventive care, in low-income adolescents [49], and has been directly linked to improved educational outcomes, including decreased absenteeism [50]. School based interventions that target educational outcomes themselves, such as those that seek to increase teacher involvement [51], parental involvement [52], and other forms of social and school connectedness [53], would be beneficial to this adolescent population as well. The Coordinated School Health approach seeks to address all of the above issues, and is easily customizable to the needs and resources available to individual schools. This is especially important in the U.S., as policies and regulations at the school level vary from state to state, as well as between school districts within states. Thus, multilevel interventions such as this are more achievable to implement and more appropriate to the suggested forms of intervention necessary to improve health and educational outcomes in this adolescent population.

## Strengths and limitations

For this study, children’s overall health status, BMI status, and educational outcomes were based on parental reports and may not accurately reflect the true prevalence, particularly among older adolescents [2,3,32,33]. However, the prevalence of overall health problems is consistent with those reported in other epidemiologic studies [54,55]. NSCH-based estimates of childhood obesity have also been shown to be similar to those based on measured BMI data from NHANES in past years [2,3,31], and for the present time period are in between those of NHANES [34] and Youth Risk Behavior Surveillance (YRBS) [56] estimates. Another limitation is that because of the cross-sectional nature of the NSCH, causal inferences about the relationships between BMI, household socioeconomic status, and educational/health outcomes cannot be drawn [32,33]. Additionally, as with most sample surveys, the potential for non-response bias exists for the NSCH, implying that the sample interviewed differed from the targeted child population in a systematic fashion [24,26]. Since response rates in the NSCH tend to be lower in urban areas and among low-income and ethnic-minority populations, differential non-response bias might affect (and most likely underestimate) the impact of BMI and social factors on health/educational outcomes [26]. However, the non-response adjustment to the sampling weights in the NSCH might have reduced the potential magnitude of these biases [26]. Lastly, the NSCH does not include questions on diet or nutrition and only a single question assessing parent-reported child physical activity levels, factors which have historically been associated with academic performance. Because of the weak nature or absence of these variables in the data, we were not able to control for these factors in our analyses or explore their potential roles as mediators.

Despite these limitations, the strong and consistent nature of our findings lends to the overall strength of our results. Namely, this study utilizes a nationally representative sample with a large dataset. Because of this, our results can be generalized to a larger adolescent population. Additionally, the size of the sample enables us to detect a greater effect in our analyses, providing more definitive results that can be used to inform intervention strategies and future policy decisions.

## Conclusions

This study provides greater evidence that increased weight status in children is associated with poorer educational outcomes, such as absenteeism, school problems, repeating of a grade, and decreased engagement with school. It further contributes to current research in this area by suggesting that it is not obesity alone, but also other factors characteristic to these children that likely drive the strength of the association. We initially hypothesized that health status and utilization of health care would attenuate the association between obesity and educational outcomes the most; while these variables do have an effect on the association, especially in the case of absenteeism, it appears that sociodemographic disparities have an even greater effect. Both health-related and sociodemographic factors must then be focused on as a point of intervention in order to reduce childhood obesity and improve educational outcomes. We suggest that for this population, schools are an ideal place for interventions that target obesity related behaviors, such as poor nutrition and low levels of physical activity, as the school setting can be used to improve adolescent health on an individual and environmental basis, as well as to directly improve educational outcomes. This structural approach to intervention is crucial to not only improving outcomes in these children, but also to preventing the long-term costs associated with childhood obesity and the further perpetuation of the cycle of low socioeconomic status and poor health/educational outcomes in this population.

## Competing interests

None

## Authors’ contributions

FRC led the writing of the manuscript and provided critical revisions and feedback. GKS conceived of and conducted the analyses and contributed to the writing. HSB provided critical revisions and feedback. AVW conceived of the analyses, and coordinated and contributed to the writing of the manuscript. All authors read and approved the final manuscript.

## Declarations

This article has been published as part of *International Journal of Behavioral Nutrition and Physical Activity* Volume 12 Supplement 1, 2015: The science of childhood obesity: an individual to societal framework. The full contents of the supplement are available online at http://www.ijbnpa.org/supplements/12/S1. Publication costs for this supplement were funded by the Michael & Susan Dell Foundation.
